# Pain and Shock

**Published:** 1917-04-21

**Authors:** 


The Hospital

The Workers' Newspaper of Administrative Medicine and Institutional Life, Administration, National Insurance and Health.

No. 1611, Vol. LXII. SATURDAY, APRIL 21, 1917. PRICE ONE PENNY

### PAIN AND SHOCK.

In a recent number of the *Times* there appeared an article entitled "The Analysis of Pain," a title so mysterious and so attractive that it was at once a disappointment and a relief to find that the article had nothing to do with analysis, and not much with pain. It was devoted to the recital of some striking examples of the stoical endurance of injury, even of very severe injury; but this is a very different matter from the endurance of pain. It is an old and trite observation on the field of battle that the excitement and mental stress of the fighting have the effect of diminishing, and in some cases abolishing, or at least suspending for the time being the onset of shock. The writer in the *Times*, who is evidently not a medical man, can scarcely be expected to know, what is trite knowledge to doctors, that the effect of severe injury and mutilation is not nearly so much pain as shock; and, indeed, that pain is by no means a necessary or even a frequent accompaniment of severe injury. The viscera, the internal organs, the muscles, and even the brain itself, are insensible to injury, and can be lacerated or incised without any painful accompaniment. It is an experience common to all that even superficial lacerations and incisions of the skin give rise to pain only at the moment of infliction, and though subsequent contact or friction of the injured skin cause pain, there is nothing beyond a slight soreness as long as the injured skin is neither touched nor stretched. There is, therefore, nothing wonderful in the absence of pain after even very severe injuries and mutilations. What is wonderful is the absence of shock, and this is a well-recognised phenomenon on the field of battle. Many instances are recorded in the Napoleonic wars, in particular one of a man whose arm was torn nearly off by a round shot, and who tore it completely off and tossed it into the air, shouting "*Vive l'Empereur.*" This classical case is paralleled by one related by the *Times* correspondent. "A man had his arm blown clean away. When someone pointed it out to him, *for he did not know it was gone altogether* [no italics in original], he said 'Damn!' That was all he said. He then walked three miles to a dressing station after applying a rough-and-ready tourniquet. Before he got there he was wounded again, in the head this time, but he kept on, and arrived at the hospital station laughing." There is no reason whatever to doubt the truth of this narrative, but it does not illustrate what it is supposed to illustrate—that is, the heroic endurance of pain or the strange insensibility to pain. The blowing off of the arm was an instantaneous affair, and there was no time for pain to be felt during the tearing of the skin, which was the painful part of the injury. What is miraculous is the absence of shock, for no man suffering from such surgical shock as usually attends an injury as severe as the blowing off of an arm could walk three yards, let alone three miles. Another instance given by the *Times* correspondent is that of the condemned criminal in China. "He can kneel at the end of a long line and watch the executioner doing his work right up to the time when he reaches the man next to him. Then he bows his neck and commends his soul to his ancestors with much less ado than the average European about to have a tooth out." The expression is a little obscure. The average European about to have a tooth out does not, perhaps, usually commend his soul to his ancestors, but the matter to which attention is called is that the Chinaman does not feel the anguish that a European would feel in much less agonising circumstances. This, however, has nothing to do with the endurance of the crude pain of bodily injury. We are here in another region altogether, and are discussing not the endurance of pain, but indifference to life. The Turk, we are told, sat stolidly waiting the time when his wounds could be attended to, in many cases refused anæsthetics, and sat up on the operating table smoking cigarettes while the surgeon did his work. This shows that the Turk was wanting in the horrified anticipation of operation. It does not show that he had any marvellous endurance of pain. The performance of the Turk is emulated by that of the English agricultural labourer. The gently born, highly cultured, quickly responsive nature that responds rapidly and energetically to all stimuli, responds rapidly and energetically to the stimulus of pain also. The stolid, vacuous, and unresponsive do not respond to the stimulus of pain, inasmuch as they do not feel it with anything approaching the same intensity. This is easily explicable. What is not explicable is the effect of the marvellous exultation and excitement of the battlefield in preventing or postponing the advent of shock. It would be extremely interesting to know whether, in such cases as the *Times* correspondent describes, shock ultimately occurs, and, if so, with what severity it occurs. *A priori* one would suppose that it is postponed, and that when it does at length appear, it appears with fatal intensity.

